# Implementation of guppy fish *(Poecilia reticulata)*, and a novel larvicide (Pyriproxyfen) product (Sumilarv 2MR) for dengue control in Cambodia: A qualitative study of acceptability, sustainability and community engagement

**DOI:** 10.1371/journal.pntd.0007907

**Published:** 2019-11-18

**Authors:** Muhammad Shafique, Sergio Lopes, Dyna Doum, Vanney Keo, Ly Sokha, BunLeng Sam, Chan Vibol, Neal Alexander, John Bradley, Marco Liverani, Jeffrey Hii, Leang Rithea, Siddhi Aryal, John Hustedt

**Affiliations:** 1 Technical Department, Malaria Consortium, Phnom Penh, Cambodia; 2 National Dengue Control Program, National Center of Parasitology, Entomology, and Malaria Control, Phnom Penh, Cambodia; 3 Malaria and other Vector-borne and Parasitic diseases, World Health Organization, Phnom Penh, Cambodia; 4 MRC Tropical Epidemiology Group, Department of Infectious Disease Epidemiology, London School of Hygiene & Tropical Medicine, London, United Kingdom; 5 Department of Global Health and Development, London School of Hygiene & Tropical Medicine, London, United Kingdom; National Center for Atmospheric Research, UNITED STATES

## Abstract

**Background:**

In Cambodia dengue vector control activities are focused on larviciding with temephos and pyrethroid based adulticide sprays to which *Aedes* have been shown to be increasingly resistant.

A cluster randomized trial assessed the impact of using biological control tools (guppy fish, pyriproxyfen (PPF), and Communication for Behavioral Impact (COMBI) activities in combination), which would be used in a value comparison to traditional chemical control tools. Given these new intervention methods, a qualitative assessment was designed in order to represent the quality of understanding, acceptance, and implementation by participants.

**Methodology/Principal findings:**

A total of 103 participants in 12 Focus Group Discussions (FGDs) and nine In-Depth Interviews (IDIs) were included in the study. The majority of participants in intervention villages (50 out of 80) preferred guppy fish over other vector control methods due to ease of use and rearing, quick reproduction and propensity to eat larvae. A substantial number of participants (11 out of 40) in intervention villages with PPF favored it due to long-lasting effectiveness, lack of smell and easy maintenance. Participants showed high demand for both interventions and were willing to pay between 100–500 riel (0.03–0.13 USD). Nearly all participants perceived that the interventions resulted in a reduction in *Aedes* mosquitos (both adults and immatures) and dengue cases. The presence of larvae in the water despite the use of PPF was a source of concern for some participants, although this was overcome in some cases with proper health education through health volunteers. Interpersonal communication through health volunteers was the most favorite method of transmitting prevention messages.

**Conclusions/Significance:**

The community led COMBI strategy resulted in high acceptance and perceived effectiveness of the interventions in target villages. Health volunteers are an effective and accepted channel of communication to engage communities, disseminate information and promote behavioral change at the household and community level. If shown effective through corresponding entomological surveys, the interventions should be continued and further strengthened to ensure they are accessible, available and affordable.

## Author summary

Dengue is one of the most rapidly spreading mosquito-borne viral diseases in the world and is caused by bites of infected *Aedes* mosquitoes. Dengue infection is a systemic and dynamic disease with a wide clinical spectrum that includes both severe and non-severe manifestations. In some cases dengue can lead to death. Cambodia has one of the highest per-capita incidence rates. Without a cure or routinely available vaccine, dengue control relies largely on reduction and avoidance mosquitoes. In Cambodia dengue mosquito control activities are focused on larviciding with temephos and pyrethroid based adulticide sprays to which *Aedes* have been shown to be increasingly resistant. The current qualitative study was designed to better understand the community acceptance and perceptions of biological controls that would overcome insecticide resistance for consideration in dengue control strategies for the future. Two control methods were deployed in different size water containers: (1) guppy fish to predate mosquito larvae in large containers, and (2) a slow-release larvicide (pyriproxyfen) product in small containers. A total of 103 people participated in 12 FGDs and nine IDIs. Both methods were generally seen positively. The advantages of guppy fish were their ease of use and rearing, and evident propensity to eat larvae. The advantages of pyriproxyfen was its long-lasting effectiveness, lack of smell, and easy maintenance. Interpersonal communication through health volunteers was the most preferred method of transmitting prevention messages. Both mosquito control interventions should be considered in future projects in Cambodia.

## Introduction

Dengue incidence has been dramatically growing around the world with an estimated 390 million dengue infections per year [1] most of which are likely undetected by most surveillance systems [2]. Without a widely available vaccine, dengue control has been relying on vector control interventions focused on eliminating larval habitats [3]. The need for community engagement in vector control becomes more important as the presence of *Aedes* aegypti hinge on human behavior. Therefore, simple, cost effective, community-led and sustainable strategies to reduce vector abundance are essential to control dengue [4].

In Cambodia, dengue vector control activities have been focused on the use of larviciding with temephos (e.g. Abate) and adulticides (e.g. thermal fogging with pyrethroids) [5]. However, recent studies have shown mosquitoes to be resistant to both currently used larvicides (temephos) and adulticides (pyrethroids) [5,6]. Alternative control methods have been evaluated in Cambodia including pilot studies which suggested that guppy fish could be used as an appropriate, effective and acceptable biological method to control mosquito breeding [7–9]. Additional studies using larvivorous fish for vector control have been evaluated in the past with varying degrees of success, although no cluster randomized controlled studies had been completed [10]. Pyriproxyfen (PPF), a juvenile hormone analogue that interferes with the metamorphosis of juvenile *Aedes* mosquitoes preventing their development into adults has also shown promising results and can be integrated in smaller containers where it is difficult for guppies to live [11,12]. Therefore, a cluster randomized study was designed to evaluate the effectiveness of these alternative interventions in Cambodia [13].

Successful implementation of these interventions, however, is not only dependent on their entomological efficacy. As other vector control measures biological and chemical control agents are also highly reliant on community acceptance[14], and fear of water contamination and toxicity has been reported in Cambodia as a barrier to community adherence to these tools [5,8,15]. Hence, the need to engage communities as active partners in planning, implementation and monitoring and evaluation activities of vector control programs [16–18]. This study used an Integrated Vector Management (IVM) approach to ensure community engagement and stakeholders involvement in designing and implementing dengue control strategies [19]. Communities take the lead in the project design, planning and decision making which helps to create community acceptance, ownership and ensure sustained community participation in the dengue program [20–22].

Culturally appropriate, well-informed and multipronged behavior change communication approaches as part of IVM are very important to increase awareness and address the misperceptions on dengue infection and dengue control tools in the communities [23–25]. Communication for Behavior Impact (COMBI) consists of approaches that ensure community participation in the design, planning and implementation and develops well-informed communication strategies, messages and materials. These messages are reinforced using multipronged channels to reach out to the different target audiences such as community members, farmers and forest workers. COMBI is a strategy that has been used successfully in other regions for dengue prevention and control [26,27].

In order to understand how well these behavior change interventions were understood and acted upon by the community, a qualitative assessment was designed. The assessment was conducted to (1) assess the community’s knowledge, attitudes and practices around vector borne disease prevention and health-seeking behaviors; (2) explore the community’s perception and acceptability of guppy and PPF use; (3) explore enabling factors and barriers for use of guppy fish and PPF; and (4) understand the community’s willingness to pay for guppies, PPF, and other vector control methods.

## Methods

### Study site

This qualitative study was conducted during August 2016. The study site was that of a larger cluster randomized trial [13] and included 30 clusters in two operational districts (ODs) within Kampong Cham province. Kampong Cham is situated along the Mekong River at an elevation of 20–30 meters above sea level. In Khmer language, Kampong means harbor and Cham refers to the ethnicity of the population living in the province, which means ‘Harbor of Cham people’. This province is around 127 kilometer far from the capital city of Phnom Penh. The climate of the province is humid and tropical. According to the General Population Census of Kingdom of Cambodia 2019, the estimated population of the province is 895,763 which makes it 7^th^ most populous province of Cambodia. The population density is 197 per square kilometer. The population speaks Khmer language and majority is involved in agriculture.

The clusters were randomly assigned on a 1:1:1 basis to one of two intervention arms and one control arm. Kampong Cham province was selected as it has one of the highest dengue incidence rates of 1.6 cases per 1000 people in Cambodia and the environmental characteristics are similar to most dengue-endemic areas of Cambodia (personal communication, Dr Hai Ra, 2016). The dry season lasts from December to April, the light rain season from April-July, and the heavy rain season from August-October.

### Participants

A total of 12 FGDs were held with male and female community members (46 male, 48 female) in the intervention and control clusters (See Table 1). Two further FGDs were conducted with health volunteers. A total of nine in-depth interviews were held with health volunteers, CNM staff, health center chiefs and village heads to explore experiences in more detail. There was no refusal observed from any participants to partake in the FGDs or IDIs.

**Table 1:** Number of in-depth interviews and focus group discussions.

Area	Types of respondents	# of IDIs	# of FGDs	Male	Female	Total Number of participants
**Intervention area 1** (Guppies+Pyriproxyfen (PPF)+Communication for Behavioral Impact (COMBI)	Community members		4	16	16	32
	Volunteers		1	2	4	06
	CNM staff	1		1	0	01
	Health Center chief	1		1	0	01
	Village head	2		2	0	02
**Intervention area 2** (Guppies+COMBI)	Community members		4	16	16	32
	Volunteers		1	4	4	08
	Health Centre chief	1		1	0	01
	Village head	2		2	0	02
**Control (no intervention)**	Community members		2	8	8	16
	Volunteers	2		1	1	02
Total participants FGD				46	48	94
Total participants IDI				8	1	9
**Grand total**		**9**	**12**	**54**	**49**	**103**

### Interventions

Study arm 1 receives all three interventions i.e. guppy fish, PPF matrix and COMBI, while study arm 2 received only guppy fish and COMBI activities, and arm 3 received only the standard vector-control activities from the Ministry of Health. PPF was used in matrix form (Sumilarv 2MR). The disks were cut to sizes appropriate for the volume of each container [13]. A well-informed COMBI strategy was developed based on the formative research. The priority behaviors, target audiences, key messages and communication channels were identified based on the formative research findings (See Table 2).

**Table 2:** The COMBI strategy framework.

Priority behaviors	Target audience	Existing behaviors	Key messages	Tools/channel of communication
Dengue Knowledge, attitude and risk perceptions	Primary and secondary: - Care givers/ - Community members - Volunteers - Health center staff	- Most of the community members were aware of the sign and symptoms and mode of transmission of dengue	The bite of an infected “Tiger” mosquito (*Aedes aegypti*) causes dengue - “Tiger” mosquito breeds in the clean waters jars, small containers or anything that hold water around your households- The “Tiger” mosquito bites during early morning and early evening	Interpersonal communication (IPC):- Health education sessions by village health volunteers (VHV) - Household visits by VHV Local media: - Loudspeaker, *tuk tuks* announcements - Dengue songs, IEC materials made by local volunteers: - Flip charts - Posters - Pamphlets - Songs CD
Health seeking behavior	Primary and secondary: - Care givers/ - Community members - Volunteers - Health center staff	- Many community start with self-medication - Many go to drug shops or private providers first for treatment - Majority of community members adopt ‘wait-and-see’ strategy for treatment and wait until the symptoms get worse	- Seek early diagnosis and treatment, if you have high fever, nausea, headache, and body aches - If you have fever, nausea, body aches, do not take any medicine by yourself, go to the health centre for proper diagnosis and treatment	Interpersonal communication (IPC): - Health education sessions by VHV - Household visits by VHV Local media: - Loudspeaker, *tuk tuks* announcements - Songs IEC materials made by local volunteers: - Flip charts - Posters - Pamphlets - Songs CD
Preventive measures Guppy fish Pyriproxyfen (PPF) Environmental cleaning	Primary and secondary: - Care givers/ - Community members - Volunteers - Health center staff	- Majority do not clean and cover their water jars - Majority do not clean their household environment - Majority do not use guppy fish - No one knows about Pyriproxyfen - Majority do not talk to their neighbors about their environment	- Clean and cover your water containers to avoid larvae breeding - Empty jars on weekly basis to avoid larvae breeding - Always put guppy fish in your large (>50L) water jars to kill the larvae - Put PPF in small containers <50 litres to stop the growth of the larva - Use insecticide net during day and night to avoid mosquito bite - Talk to your neighbors about cleaning the environment	Interpersonal communication (IPC): - Health education sessions by VHV - Household visits by VHV Local media: - Loudspeaker, *tuk tuks* announcements - Songs IEC materials made by local volunteers: - Flip charts - Posters - Pamphlets - Songs CD

The messages were transformed into sketches or pictorials by the local health volunteers to ensure the ownership and foster sustainability. The local health education materials including pamphlets, posters and flip charts were prepared based on these sketches and messages which were disseminated to the communities through health volunteers, megaphone announcements and songs. The key messages were consistent across the materials i.e. pamphlets, posters and flip chart to ensure reinforcement. The songs on the key dengue messages were written and recorded by local entertainment artist in Khmer language, and disseminated through mega phone by mobile *tuk tuks* which are popular motorized rickshaws. The health volunteers were trained in communication and community mobilization skills to ensure effective message delivery and active community engagement in the project. The Centre for Parasitology, Entomology and Malaria Control (CNM), Operations District, Health Center staff and Malaria Consortium jointly organized and facilitated the two-day training at the District Hall in Prey Chhor. The total trial period for the interventions was 11 months. The qualitative study was conducted the month after the COMBI activities ended. Table 3 shows the village assignments to either intervention or control arms.

**Table 3:** Intervention and control village assignment.

Study Arm	Interventions	Study villages
Arm 1	Guppy + Pyriproxyfen + COMBI	Ampil Chrum, Romeas
Arm 2	Guppy + COMBI	Rong Kor, Banteay Roeng
Arm 3	Control—standard vector control activities	Tropeang Kork

### Data collection

Focus group discussions were conducted with community members and health volunteers to understand the preferences and acceptability of interventions (guppy fish and PPF). A topic guide with major themes and key probes were developed to conduct the in-depth discussion. Various participatory tools such as free listing, pair-wise ranking and Likert scale were used to assess the preferences and generate the discussion. The pair-wise matrix was used to understand their perception about severity of major health issues. A list of existing diseases, including vector borne and chronic, was constructed and placed in a matrix table drawn on a flip chart. Each disease is compared to the other disease individually and then the number of times it was chosen is summed. The disease with the largest sum is deemed to be the most important disease perceived by the participants. The Likert scale was used to identify the most liked vector control interventions in the community. A free list of all existing interventions was constructed by the participants. A set of pre-made cards with sketches of all interventions (one sketch per card) were handed over to the FGD participants. A flip chart with eight rows, one for each participant and three columns 1) like the most 2) neutral 3) disliked the most were developed and put on the ground. Each participant was given the set of cards with the existing interventions and asked to put one intervention card in one category according to their choice. All activities including free listing, pair-wise ranking and Likert scale were conducted during the FGDs, transcribed and analyzed. The observation visits were also carried out to the participants’ households after the FGDs to validate the use of guppy fish and PPF in their containers. The participatory methods helped stimulate engagement with the participants and encouraged focused discussion on the key issues under investigation [28].

A semi-structured topic guide was used for in-depth interviews. The interviews were carried out with CNM staff, health center chiefs, village heads and village volunteers to understand the access, maintenance and sustainability of these interventions.

Purposive sampling technique was used, based on potential participants’ availability, special knowledge, interest, and willingness to participate in the study. A total of 54 men and 49 women participated in the study. All participants were adults over 18 year of age; the age groups of men and women were between 29 to 62 years and 25 to 61 years, respectively. The participants were recruited a day before the actual data collection with the help of village health volunteers. The participants in FGDs were purposively sampled to capture diversity in gender, age groups, roles in the communities. The numbers of interviews and FGDs were chosen with the aim of reaching data saturation. To minimize inhibitions and encourage open debate, FGDs were homogenized with regard to age and gender of the respondents. At least two focus group discussions were conducted with each category of respondents to triangulate the findings. The data was triangulated by data collection teams, methods i.e. interviews/FGDs, participatory tools and participants (gender). However, the participants were not involved in the data checking. FGDs were conducted in an accessible yet private location in each community, such as a health center, volunteer’s house or monastery. A pack of two bars of soap, two drinking water bottles and a small piece of cake (cost of 1 USD/person) was given to the study participants at the end of the FGDs and IDIs. This served as a token of appreciation for their time and participation in the study.

Topic guides for FGDs and IDIs were developed in English and translated into Khmer language to facilitate the local interviewers in data collection. The topic guides were pre-tested and finalized based on the respondents’ feedback. Face to face FGDs were conducted by two teams of three data collectors (one facilitator and two note takers) supported by one senior supervisor from Malaria Consortium. IDIs were carried out, one-on-one, by a facilitator who simultaneously took the notes during the interview. The data collection team was comprised of four male and two female members. IDIs and FGDs were conducted at neutral common places such as volunteer’s houses, health centers and monasteries. Only selected participants were involved in the data collection. The interviews were conducted in private settings to avoid the interference of non-participants. An average focus group discussion took around 2 hours while an IDI lasted for 1 hour. The team had sufficient field experience in qualitative data collection. Interviewers were hired from outside of the study areas to avoid any bias. A two-day refresher training was held covering facilitation, moderation and probing skills, note taking skills, transcription of the interviews and research ethics.

### Analysis

The data collection team took detailed notes and also used digital voice recorders (Sony Digital Voice Recorder, ICD-PX312) with prior permission to record the interviews. A daily feedback session was held with the study team to discuss the process, issues/gaps, interesting information, data saturation and emerging themes. The topic guides were adapted based on the emerging themes. The FGDs and IDIs were transcribed verbatim in Khmer language by the study team on the same evening to avoid any information loss or recall bias. There were no major concerns in the transcription to go back to the community for any further confirmation. The FGD and IDI transcripts were translated into English by two experienced translators who previously participated in similar studies. The translated transcript were shared with the research team for their review to ensure the quality of translation. The coding and analysis was done by one person, however, the key results were first shared with the study team to validate the findings.

The Framework Approach [29,30]was carried out based on the following steps: Familiarization—key themes were identified during a meticulous review of the transcripts; Thematic framework construction—themes deriving from the study objectives and other key issues that emerged from the data were identified and used to assemble a coding/thematic framework in an Excel spreadsheet; Indexing—the data were coded according to the thematic framework by target group and re-organized into sections under each theme; Interpretation—each thematic area was compared between respondent groups, similarities and associations between themes were identified and findings were interpreted.

Key quotation were identified and separately organized under each theme to support and validate the findings.

### Ethical consideration

Ethical clearance for this trial has been received by the Cambodian National Ethics Committee for Health Research (ethics reference number 0285). Additionally, ethics approval was received from the London School of Hygiene and Tropical Medicine Observational / Interventions Research Ethics Committee (ethics reference number 8812). All the study participants were of age 18 or above. A written informed consent was taken from all participants before the start of the IDIs and FGDs.

## Results

### Findings

Four core analytic themes and 12 subthemes (See Table 4) were interpreted within the data including: (1) Dengue Knowledge and Attitudes, (2) Health Seeking Behavior, (3) Vector Control Perceptions, (4) Project Perceptions. Acceptability, sustainability, and community engagement were not addressed explicitly as a theme, but rather included throughout the core themes and subthemes.

**Table 4:** Key themes and subthemes identified in FGDs and IDIs.

Core Theme	Dengue Knowledge, Attitudes, and Perceptions	Health Seeking Behavior	Vector Control Perceptions	IVM Project Perceptions
**Sub Theme**	Dengue Knowledge and Attitudes	Health seeking preferences	Existing vector control measures	Behavior Change
	Risk Perceptions	Key barriers to healthcare	Guppy Fish	COMBI Activities
			Demand and sustainability of guppies	
			PPF	
			Demand and sustainability of PPF	
			Vector Control Preferences	

### Dengue knowledge, attitudes, and perceptions

#### Dengue knowledge and attitudes

The knowledge of dengue transmission was very high among the community members in both intervention and control villages. The majority of the community members, both male and female, mentioned that only a bite of a *mos khla* or tiger mosquito causes dengue. Also, many mentioned the scientific name (*Aedes aegypti*) of the mosquito vector.

“The *mos khla* [tiger mosquito] causes dengue. If there are no larvae or tiger mosquitos, there will be no dengue as well”. FGD, female, community member, Arm 2.

“A person gets dengue when he/she is bitten by a tiger mosquito.” FGD, female, community member, Arm 1.

“The dengue disease is caused by a tiger or *Aedes* mosquito who bites during the day time”. FGD, male, community member, Arm 3.

The community members from intervention areas also had a very good understanding of biting times of the dengue vector. Most of the community members mentioned that *Aedes* or tiger mosquito bites during the day time.

“The tiger mosquitoes bite in the morning and early evening. They bite between 9 am and 4 pm.” FGD, female, community member, Arm 2

“The dengue mosquito bites you from 9:00 am until late afternoon". FGD, female community member, Arm 2.

The majority of the participants from intervention villages knew the main dengue signs and symptoms. While most identified high fever as the main symptom, they were also able to point out headache, fatigue, vomiting, rashes and skin bleeding as dengue signs and symptoms. Many also noted that convulsion, bleeding from nose or skin and black stool are symptoms of severe dengue.

“Dengue causes fever, headache and skin rashes”. FGD, female, community members, Arm1.

“Convulsions, bleeding from gums and nose and black stool are the signs of severe dengue.” FGD, female, community member, Arm 2

The community members from the intervention and control villages mentioned that dengue is widespread during the rainy season. This lasts from May till November. They mentioned that mosquitoes are in abundance and breed everywhere especially in small ditches, ponds and stagnant water during the rainy season.

“Dengue is common in rainy season that starts in May. The standing water breeds *dong kao toeuk* [larvae] and *Aedes* mosquitos which cause dengue”. FGD, female, community member, Arm 2.

Many community members especially in intervention villages mentioned that dengue mosquitos breed inside or near the households. The mosquito breeds in water containers, cans, coconut shells and tires.

“Mosquitos breed in bottles, cans, water containers, old tires and in the standing water around the household”. FGD, male, community member, Arm 2.

#### Risk perceptions

The pair-wise ranking was carried out to identify the most important health problems in the target communities. Pair-wise ranking confirmed that the majority of the community members from intervention and control villages believe that dengue is one of the most common health problems. They considered dengue as dangerous and were concerned patients may die if not treated in time. The next most mentioned illnesses were high blood pressure and diarrhea.

“My nephew died of dengue because his parents did not take him to the hospital in time.” FGD female, community member, Arm 2.

“Dengue is a dangerous disease. I have to rush to the hospital immediately when my child gets sick”. FGD, female, community member, Arm 1.

Most community members from the intervention villages perceived children between 1–12 years of age as the most at-risk group for dengue. They also noted that in the past dengue mostly affected the children, however, now young and old people are also at risk of contracting dengue. For participants, the main reason they felt children are more vulnerable to get dengue is because they cannot protect themselves from the mosquito bites.

“It [dengue] mostly affects the children between ages 5 to 8 years. However, young or old people can also get dengue now-a-days”. FGD male, community member, Arm 2.

The health volunteers also confirmed that children are the most high-risk group for dengue in these communities.

“Mostly, children under 15 get dengue. It is more common in the children because they play in the dark places without any protection”. FGD, village health volunteers, Arm 2.

Several community members stressed the economic implications of dengue, mentioning that it wastes money, resources and productive time when seeking care. They stated that dengue affects their livelihood and earnings and wastes their productive time during illness as they need to take care of the patients for several days.

“We have to go to rice field every day. But if our child gets sick, we have to stay home and look after him for 7 days. It wastes our productive time.” FGD, female, community member, Arm 1.

### Health seeking behaviors

#### Health seeking preferences

The focus group discussions revealed that majority of respondents starts with self-medication when they get sick. They adopt a wait-and-see strategy and if there is no improvement in their symptoms after 2–3 days, they start looking for other available options for treatment. When deciding on health care options, participants usually chose public health system structures like health centers and district/provincial hospitals, but calling private providers was also mentioned as a strategy to avoid long waiting times in hospitals.

“We buy medicines from a village shop or pharmacy and wait for 2 to 3 days to see results. If we do not feel better, we go to Kampong Cham for treatment.” FGD, female, community member, Arm 3.

Traditional healers, herbal treatments and fortune tellers were also identified as ways to treat or identify the disease.

“We still use the traditional herbs such as *neem* [an indigenous tree], to cure dengue fever. We squeeze the neem leaves and drink the neem juice to treat the dengue fever.” FGD, male, community member, Arm 1.

“If our kids get sick we go to fortune teller first to guess the disease. If the kids do not feel well, we will take them to hospital. FGD, female, community member, Arm 3.

#### Key barriers to healthcare

Lack of financial resources, lack of transportation, distance to health facilities, long waiting time (queues) at the hospitals and impolite attitude of some health care providers were considered the main barriers to receiving dengue diagnosis and treatment services from the public health facilities in both intervention and control villages.

“Lack of financial resources, bad road conditions and lack of transport are main difficulties to seek treatment from government hospital.” FGD female, community member, Arm 2.

“The key difficulties include lack of money, lack of transportation and long waiting time [long queues] which waste our productive time.” FGD male, community member, Arm 1.

### Vector control perceptions, acceptability and demand

#### Existing vector control measures

The free listing exercise was conducted during the FGDs to come up with the variety of existing vector control measures. The existing vector control measures mentioned by the intervention communities can be divided into three categories: environmental measures (e.g. use of bed nets, wearing long sleeved clothes, cleaning/covering water jars, and burying tires and coconut shells), biological measures (e.g. guppy fish and *Bacillus thuringiensis israelensis* (Bti), and chemical measures (e.g. PPF, temephos, chemical sprays, repellents, lotions and mosquito coils). The guppy fish was known as *Trei pram pei por* [seven colored fish] in these communities. ‘PPF’ was used as loan words in Khmer.

“I use *trei pram pei por* [seven colored fish] or guppy fish to eat larvae, cut down some plants, burn garbage to make fire, make my children wear long sleeves and use bed net to avoid mosquito bites.” FGD, female, community member, Arm 1.

After free listing, the Likert scale activity was carried out to identify the most preferred vector control measures. These exercises helped generate discussions on the various vector control methods during the FGDs.

In the control villages the majority of community members mentioned bed nets, long sleeved clothes, chemical sprays, mosquito coils and temephos as the main prevention methods for vector control. Among the additional vector control measures mentioned were: environmental management, burning old tires, cleaning water jars and removing tiger mosquito habitats or breeding places. By contrast, many complained about undesirable side effects and costs of chemical products.

“We use bed nets and wear long sleeved clothes to prevent mosquito bites and dengue”. FGD male, community member, Arm 3.

“We dislike chemical spray as it is expensive, can ward off mosquitos for only a short period of time and is harmful for the health of children.” FGD female, community member, Arm 3.

A few community members in the control villages were aware that the guppy fish can be used as a vector control measure; however, most had not seen any before. Some people mentioned that they use the *Kranh fish* [a kind of rice field fish] in their water jars to control larvae.

“I do not know about guppy fish. It is hard to find even *Kranh* fish in these communities”. FGD female, community member, Arm 3.

None of the community members in the control group were familiar with the PPF method.

#### Guppy fish

The guppy fish known as *trei pram pei por* [seven colored fish] had high acceptance in the intervention communities and was referred as the most preferred vector control method. The main reasons for preferring guppies over other methods were their aesthetic attractiveness, ease of use and rearing, quick reproduction, sustainability, propensity to eat larvae and lack of bad smell.

“We liked guppies as they eat larvae, clean the water from larvae and eliminate tiger mosquitoes.” FGD, female, community member, Arm 2.

“The guppies eat larvae and reduce the mosquitos. There were a lot of mosquitos when we did not have guppies. Now guppies have decreased half number of mosquitos compare to the past”. FGD male, community member, Arm 1.

Additionally, some community members perceived the guppy fish as the natural (biological) and less harmful method for larvae control.

“Guppies are not harmful as they are a natural method. They don’t have any bad smell and do not pollute the water that we use.” FGD, male, community member, Arm 2.

A few community members also compared guppies with other methods such as temephos and found guppies better with regards to smell or water contamination.

“The guppy fish does not smell bad. We can use the guppy water whereas in case of Abate [synonymous with temephos], we cannot use water as Abate smell very bad”. FGD, male, community member, Arm 1.

Communities’ preference for guppy fish was also corroborated by health center staff and volunteers.

“We distributed Abate in the previous years. Now we have stored guppies in the health centers. The community members prefer guppies more than Abate.” IDI, health center staff

The CNM staff also noted that community members prefer and like guppy fish more than they used to do in the past. They attributed this change to the focused COMBI and regular distribution of guppies by the volunteers.

“I think that they [community members] have changed their behavior. In the past, they disliked guppies but now they use and like them. When they don’t have guppies, they try to find them.” IDI, CNM staff.

The health center staff mentioned that in the beginning some community members were skeptical about guppy fish and reluctant to put them in their water jars considering they might have a bad smell. However, after health education visits from the health volunteers they agreed to put guppy fish in their water jars and have not had concerns since. Despite the work of the health volunteers a very small number of households still refused to use guppies as they considered it smelly and dirty.

“In the beginning, some people didn’t understand well and didn’t allow us to put guppy fish in their water jars. However, when they saw that guppy fish eat larvae, and helped avoid mosquito bites and protect them and their children from dengue, they agreed.” IDI, health center.

Some causes for guppy mortality and loss were identified by the community members, namely predators like lizards (e.g. geckos) and green frogs, children as they played ‘fish fights’ with guppies, excess rain in rainy season as guppies were flushed out of water due to overflow, household water use, accidentally scooping out guppies when bathing at night, and death from direct exposure to sun during the dry season.

“We have to be careful of lizards and green frogs that eat guppies. We need to cover the containers to avoid frogs and lizards to protect guppies”. FGD, female, community member, Arm 2.

“We cover the water jars. Because the color of the guppies is so nice that children steal them. Sometimes, they take them for fish fighting.” FGD, female, community member, Arm 2.

“The guppies died in water jars which were placed under the sun without cover. In rainy season, jars were full of water and guppies spilled out of the water and died.” FGD, male, community member, Arm 1.

A few also mentioned that guppy fish died when community members fed them too much.

#### Demand and sustainability of guppy fish

Easy maintenance, rearing and reproduction were perceived as key sustainability factors for guppy fish by the community members in intervention villages. Most of the community members mentioned that guppy fish are easy to maintain and therefore are a sustainable vector control measure.

“The seven colored guppy fish can never end, it gives birth and multiplies which can be used and shared with other community members consecutively.” FGD, health volunteers, Arm 2.

Additionally, there was a strong commitment from volunteers to continue COMBI and guppy fish in their villages. They mentioned that they will continue breeding guppies in their villages and keep sharing it with other community members to avoid mosquito and dengue cases.

“We want to maintain guppy fish forever. We are aware of the benefits of guppy fish as there is no outbreak. They are beautiful and effective as well.” FGD, health volunteers, Arm 2.

Many community members showed their willingness to buy the guppy fish after the closure of the project. They inquired about the possible places to buy guppies for their continuing use. Most community members were willing to pay between 100–500 riel (0.03–0.13 USD) for a pair of guppy fish.

“If guppies can eliminate the disease, I will buy even if it cost me 1000 riels (0.25 USD) per guppy. The cost of the treatment is more expensive than that of the guppies.” FGD, female, community member, Arm 1.

The health center staff noted that there is a lot of demand for guppies not only in the intervention villages but also beyond the intervention villages. They mentioned that many people from other villages come and request guppy fish as they have heard good things about guppy fish from this project. The health center staff mentioned that as they already have the infrastructure for distribution (i.e. guppy fish jars), therefore they will be happy to continue rearing fish along with community health education.

“People have realized the benefits of the project as they observed very few sick children in the intervention villages (this year). Therefore, people from neighboring villages come and request guppy fish for their villages.” IDI, health center staff

#### Pyriproxyfen (PPF)

Many (11/40) community members (both men and women) from the PPF clusters preferred PPF over the guppy fish and other vector control measure and found it effective to reduce the mosquitos from the community. The majority of the PPF users understood that PPF stops the growth of larvae to become an adult mosquito and transmit dengue. Most of the community members in the PPF cluster knew how to use the PPF and were aware how long the PPF chemical lasts.

“Before the use of PPF, whenever we would open the jar covers, plenty of mosquitoes swarmed out of the jar. Since we use it, when we open jars, there are no mosquitoes flying out. There are larvae still in the water but they cannot fly.” FGD, female, community member, Arm 1.

Some also suggested that use of PPF is even more convenient than guppy fish as it requires less hassle during cleaning or changing of water.

“It is easy to clean the water jar with PPF. We can take it [PPF] out, clean the water jar and put it back. However, in case of guppy fish it is a different story.” FGD, male, community member, Arm 1.

Most of the community members did not have any fear of chemical or side effects of PPF. However, some of the female community members mentioned that initially they were concerned about the toxicity but later on volunteers alleviated their fears with focused health education. The health center staff also confirmed that in the beginning some community members were worried about the PPF use but later on understood the benefits and started using it regularly.

“In the beginning, I was afraid of poison. However, since the village volunteers have explained well, I stopped worrying and started using PPF.” FGD, female, community member, Arm 1.

As per the project design, the guppy fish were promoted for bigger containers (>50 liters) and PPF for smaller jars (10–50 liters) which might have led some people to think that PPF can only be used in small jars which was not entirely correct. Therefore, many people in the project misperceived that PPF is only appropriate for smaller jars.

“I don’t prefer PPF over guppies as it can only be used for smaller water jars. I have only big containers where I can’t use it.” FGD, female, community member, Arm 1.

A small number of community members showed concerns over the presence of larvae [*Dong kao toeuk*] in the water despite PPF use. They considered that the presence of larvae may contain the virus and potentially spread the disease.

“We know PPF works well as fewer mosquitos around, however, we are afraid the presence of larvae may contain parasites that can spread the disease.” FGD, male, community member, Arm 1.

Another issue with PPF was the nuisance of children who considered it a toy to play with. Many community members expressed that children stole PPF from their containers and destroyed it.

“It’s easy to put PPF in water but we had to prevent it from the reach of children who take it out and play with it.” FGD, male, community member, Arm 1.

#### Demand and sustainability of PPF

One of the major concerns of the community members especially men was the availability, accessibility and affordability of PPF after the completion of the project. Many community members inquired about its cost and availability in the local market. A few community members also shared their willingness to buy the PPF if it were available in the local market at an affordable price. Many were willing to pay between 200–500 (0.05–0.13 USD) riels for a piece of PPF.

“The other day, I asked my wife to buy PPF from the market. She informed that it is not available yet. It should be available in the market so that we could buy it after the project.” FGD, male, community member, Arm 1.

A few community members compared PPF with other chemicals such as temephos and found it more desirable. The main reasons were its lack of smell and ability to not to affect the quality of water, as opposed to temephos which was perceived to have a bad pungent smell.

“We are not afraid of insecticide or bad smell of PPF. There is no bad smell at all. Abate, which is also a chemical, has very bad smell”. FGD, female, community member, Arm 1.

#### Vector control preferences

The community members articulated that both interventions (guppy fish and PPF) have different benefits depending on the individual’s use and needs (See Table 5). Many liked both interventions, but when it comes to comparison the majority (50 out of 80) preferred guppy fish as they were perceived as a natural, sustainable, and easy to use and maintain vector control method.

**Table 5:** The pros and cons of using Guppy fish and Pyriproxyfen.

Interventions	Pros	Cons
Guppy fish	• Attractive and colorful • Considered natural/ biological method to eliminate larvae • Easy to use, feed and maintain • Sustainable as reproduces easily • Do not pollute water, no bad smell, no bad odor • People use the water with guppy fish for cooking and washing without problem • Suitable for bigger containers of more than 50 litres • Cheap as one can continue breeding at home	• Children steal them to play ‘fish fighting’ • Predators like lizards and green frogs eat guppy fish • Difficult to maintain in rainy season as spill out from the jars and die • Cannot be used in small jars • Requires more hassle when cleaning the jars • A few believe it pollute the water
Pyriproxyfen (PPF)	• Effective for longer period i.e. for 6 months • Stops the growth of larvae • Easy to use and maintain • Easy to cut into small pieces to fit in various containers from 10–50 litres • No bad smell or odor • No fear of chemical • Easy to clean containers when using PPF • No need to worry about frogs, lizards when use PPF	• Larvae are still present in the water despite PPF use • Some people suspect PPF is not working when they see larvae in the water • Children take out and play/destroy PPF matrix • Not sustainable as difficult to find after the project is over • Could be expensive if available in the market

“PPF matrix’s life is only 6 months while we can use guppies forever until they die. We prefer guppies over the PPF.” FGD, male, community member, Arm 1.

The health staff also confirmed that one of the main reasons for preferring the guppy fish over PPF was its being a natural or biological method.

“From my point of view, putting guppy fish into the jar is a very good way to prevent larvae. People like to put guppy fish in their jars more than Abate. When we put Abate or PPF in their water jars, they suspect chemicals, however, when we put guppy fish they feel good.” IDI, Health Center staff

While the majority preferred guppy fish and PPF, most community members in intervention communities disliked many existing vector control methods (See Table 6). The reasons for disliking these methods were their perceived harmfulness, price, and lack of effectiveness. The most disliked measures mentioned were fire or smoke, mosquito coils, chemical sprays, repellents, Abate, mosquito racket and electric mosquito lamp.

“We dislike smokes, repellents, mosquito coil, chemical sprays and abate. The smoke is not good as it is harmful for our health and can cause lung disease.” FGD female, community member, Arm 2.

**Table 6:** Most disliked measures (among those not in the trial, from most to least disliked).

Most disliked method	Perceived reasons
Fire or smoke to repel mosquitos	• Fire (burning wood or branches) can be used only at night. So, when it’s extinguished, mosquitoes bite again. It also darkens the house with smoke. • Harmful for children, may cause lung disease. • Causes breathing difficulties for children
Chemical sprays (aerosol)	• Expensive, it cost 14000 riels (3.46 USD). • Harmful for children.
Mosquito coils	• Toxic for children. • Mosquito coils are not effective, as mosquito come back soon after the coil is burnt.
Repellents	• Expensive • Causes skin irritation or burning feeling on the skin.
Abate	• Abate is not effective as mosquitoes are still around despite its use • Contaminate water with bad smell. • Difficult to find in the market.
Electric Mosquito Killer Racket	• Expensive, costs 12,000 riels (2.96 USD).
Electric Mosquito lamp	• Useless, expensive and adds to the electricity bill.
Bed net	• Only useful during night, but *Aedes* mosquitos bite during the day.

### IVM project perception

The majority of the community members from the intervention arms, both men and women, were aware of the project and many participated in COMBI activities such as health education sessions. Most of the community members, volunteers and health staff expressed during the interviews that the project has achieved the community participation in its activities. Most of the female community members participated in the health education sessions to learn about guppy fish and PPF. Many men also participated in the health education sessions however, most of the men were busy in farming or working out of the village and could not participate in the COMBI or health education activities.

A majority of community members from intervention villages perceived that dengue cases reduced since the project commenced, and those in control villages perceived that dengue still occurs quite often in their villages. As there was no epidemiological measure taken during the trial the actual number of cases is unknown.

“There were many dengue cases observed last year. However, we had no dengue cases this year because of the guppies. There were only influenza and cough.” FGD, female, community member, Arm 1.

“Dengue occurs very often here in the village; we see almost 10 cases per month in our village.” FGD, male, community member, Arm 3.

Similar observations were also reported by health facility staff.

“There are very few sick children in the intervention villages. Lots of people from other villages realized that this is due to our project. They (other villagers) also come to request guppy fish.” IDI, health center

#### Behavior change

It was reported by the community members and volunteers that there were clear positive changes in behaviors such as using guppy fish and PPF, cleaning water containers, and changing water on regular basis in the intervention communities. There were some positive changes reported in the health seeking behaviors of the community members especially with regards to visiting the public health facilities for diagnosis and treatment. The community members, volunteers and health center staff reported that when people get sick and suspect dengue, they visit the public health facilities.

“In the past, we did not know much about dengue. When our children get high fever, we would take them to traditional healers or gave them Chinese medicines as we thought we have done something wrong with our ancestor. Now, when our children get high fever, we take them to health center immediately as informed by volunteer”. FGD, female, community member, Arm 2.

CNM staff also reported that people have changed their behavior to start rearing and using guppies.

“In our observations during the recent field visits, I think that they have changed their behaviors. They have more attention on using guppies. In the past, they disliked guppies but now they use and like them. When they run short of guppies, they try to find them.” IDI, CNM staff.

During the entomological monitoring surveys, field teams observed that there were less mosquitoes and larvae in the water containers than before. They attributed this to the continuous awareness and distribution of guppies and PPF by the village volunteers to the intervention communities.

“We have conducted 4 entomology surveys in the intervention villages. In the first and second survey there were lots of vectors. The 3rd and 4th survey showed a decrease in the vector. I noticed that there were not so many larvae in their water jars then” IDI, CNM staff.

#### COMBI

Community members from intervention villages described an array of communication methods including posters, pamphlets, *tuk tuk* (two-wheeled carriages pulled by motorbikes) advertisements, megaphone announcements, songs, radio and TV used to disseminate dengue related information during the project. According to the majority of the participants, interpersonal communication methods through health volunteers were the most preferred and trusted source of information in the intervention communities. The main reasons for preferring health volunteers were their ability to develop quick rapport and trust and provide face to face communication and clarify the questions on the spot.

“We prefer and trust in the village volunteers. They can meet face to face and answer direct questions. In case of TV, they just talk and we listen only and cannot ask questions.” FGD, female, community member, Arm 2.

The health center staff also confirmed that village volunteers were the most preferred and effective channel of communication for the intervention communities.

“The village health volunteers conducted house visits and explained directly therefore the community understood the information very well.” IDI, health center staff

Many community members both men and women also liked the megaphone announcements through *tuk tuk* as an interesting channel of health information. They found this useful to reach out to the farmers who cannot attend the health education sessions due to their hectic agricultural activities.

“Most of us are out in the rice fields when the health education sessions are being held in the village. Therefore, information through tuk tuk was very useful.” FGD, male, community member, Arm 1.

However, most complained that *tuk tuk* moved too fast that they could not understand the messages or songs clearly.

“We want the *tuk tuk* drivers to pass the village slowly. They should stop at one place where there are lots of people in the village and disseminate the messages and songs.” FGD, female, community member, Arm 2.

Some community members mentioned that the posters and pamphlets were also useful but not sufficient quantity-wise, for the community members. The posters were small and distributed to a few people only.

“I would like to have lots of bigger size posters. Now only some houses received poster which we cannot see.” FGD, female, community member, Arm 2.

In control villages, the main sources of communication mentioned were radio, TV, posters, head of commune, neighbors or health center staff. Community members, both male and female, mentioned that there was no formal health education activity conducted by the volunteers or health staff in the last 6 months.

“There was no health education activity organized in the village for the last 6 months. They only hear messages from their neighbors, health center or radio and TV.” FGD, female, community member, Arm 3.

## Discussion

This study aimed to examine community adaptations to the use of new biological vector controls over that of previously used methods. The results suggest that there is high knowledge of dengue in both intervention and control communities. The majority of participants perceived dengue as a common health problem in their communities and correctly identified that dengue is caused by *Aedes* mosquito bites and understood biting times. Most of the community members perceived dengue as a life-threatening disease that can kill if the treatment is delayed. This could be attributed to the continuous health education activities of CNM through health volunteers and mass media at the community and school level [23].

Nevertheless, there is still a large gap between knowledge and practice when it comes to health seeking behavior, with the majority in both intervention and control villages adopting a ‘wait and see’ strategy. This most often manifests itself by spending 2–3 days at home with self-medication before seeking treatment from a public health facility which can delay proper diagnosis and treatment. The results were also similar to the quantitative knowledge, attitudes, and practice survey completed at the beginning of the project which showed that high knowledge alone did not correlate with actual practices in the same communities [31]. However, the study showed some anecdotal improvements in the health seeking practices from intervention villages, although the findings might be due to the interview bias by respondents who sometimes want to provide desirable answers rather than the actual behaviors.

Many community members considered dengue as more of an economic issue than a health issue focusing on the loss of productive time and resources which confirms the finding of a previous study [32]. Therefore, health education messages should be revised highlighting the economic benefits and incorporated in the culturally appropriate materials and channels such as posters, pamphlets, songs and mega phone announcements through *tuk tuks* to motivate and encourage people to adopt preventive measures and go for an early diagnosis and treatment.

Larvivorous fish have already been used as vector control method in many countries including Thailand and Cambodia [8,10]. The results suggest the project was successful in creating a high acceptance and demand for locally sourced larvivorous fish (guppy fish). The communities, as reported in previous studies, believed that guppy fish were effective in controlling the larvae in their jars, and reducing the number of adult mosquitoes and suspected dengue cases [33]. The data also suggest community members are willing to pay for the guppy fish if they are available at the community level. Experience from the IVM project shows guppy fish can be mass-reared easily as they can be bred year-round in containers at the village level [8]. Hence, community-based mechanisms of guppy breeding and supply deployed for free or at a subsidized cost could be considered as a strategy to ensure the sustained access and use of guppies at the community level. A subsidized cost-based guppy supply system can keep volunteers motivated and interested in rearing guppies for the longer term. Another similar study conducted in Cambodia also suggested that if it were possible to create demand for guppies through health education and promotion activities, there is a possibility of villagers raising them independently for sale [34].

If such a strategy were designed, its success would rely on advocacy and national program engagement to support health centers and village volunteers in rearing guppies to cater to the demand of the communities.

Some concerns have been raised around the safety of having guppies in drinking water due to the introduction of pathogens such as *E*. *Coli* [35]. However, Chadee et al. sampled containers which were filled with clean water before guppy introduction and were used for breeding with more than 100 guppies in one 400-liter jar. The study also found similar pathogens to those in the guppies in the streams where the fish had been sourced, suggesting drinking water from the lakes and streams in Trinidad may already be contaminated. In rural Cambodia, much of the drinking water comes from lakes, streams, or waterways which already contain guppies and other fish. A field study in Cambodia and Laos also showed that many water samples taken from 400-liter containers before and after the introduction of a maximum of 3 guppies had unsafe levels of *E*. *Coli*, however, there was no statistically significant increase in *E*. *coli* or total coliform contamination after guppy introduction [9]. More research should be done to confirm these findings and determine exactly the number of fishes that may increase the likelihood of introducing pathogens into drinking water. In recognition of the chances of accidental guppy introduction and damage to local ecosystems [36], we urge public health authorities to prevent releases into waterways and aquatic ecosystems.

PPF was also accepted by the community members in the intervention villages. Many community members used PPF in small water containers and demand remained high through the study period. One of the main positive aspects reported by the community of PPF use is that it does not contaminate the odor or taste of water. This is consistent with previous studies conducted on PPF in Cambodia [11]. However, the major reservation noted by the community in using PPF was communities’ concerns over the presence of larvae in the water despite its use. Another reported barrier of PPF use was the false assumption that it could only be used in small water containers (as the IVM project promoted guppies in larger containers) and some households lacked the associated sizes of the water containers.

Interestingly, many people compared PPF with other chemicals available in the villages (such as Abate) and perceived PPF more effective and desirable than Abate. The reasons for this included the lack of odor or taste. Participants found PPF easy to use and maintain and enjoyed the long (six months) duration of effectiveness compared to other larvicides. However, confusion still existed over the presence of larvae in the water containers and whether the PPF was actually effective. However, these concerns were able to be overcome through the use of interpersonal communication by health volunteers. Therefore, more focused messages and health education should be provided to improve understanding on the method of action and alleviate concerns of communities which use PPF in the future. Considering the high insecticide resistance patterns identified in Cambodia [5,6,37] and in the region [38–40] in addition to the overall negative perception to Abate related to odors produced and toxicity, alternatives to Abate should be considered. PPF should be considered as one valid alternative considering the acceptability of the controlled release product used in this community.

The IVM project was able to increase community participation using the COMBI approach. The increased community participation in the guppy and PPF interventions could be attributed to the well-informed COMBI strategy, tailored messages, and activities developed based on the formative research, trained village volunteers, locally developed health education materials and use of preferred channels of communication to relay the health messages. As demonstrated in another study [41], the rapport of the local volunteers, closer interaction with community members and distribution of new effective interventions (such as larvivorous fish and PPF in this case) resulted in better understanding and stronger community participation in the IVM project. The qualitative assessment validates some previous study findings that the COMBI interventions based on the formative research were effective in mobilizing communities to establish and maintain the newly introduced interventions [42]. In the IVM project, the messages and health education materials such as leaflets, posters, songs, broadcast from *tuk tuk* were designed and developed (including messages and pictorials) by the community members and volunteers which had strong ownership by the community. The use of *tuk tuks* to broadcast songs and messages was appreciated, however communities would have preferred longer use at each village. The *tuk tuk* drivers need to be trained and sensitized on the proper implementation of the broadcast strategy. Another gap in the COMBI strategy was the lack of mobile technology to disseminate messages. The use of mobile technology can be further explored to reinforce the key messages and send reminders to community to perform certain behaviors such as changing of water in the containers.

Due to the limited financial resources, fewer number of FGDs were conducted in the control arm which might affect the comparison with the intervention arm. The study was conducted in one province only which might limit the relevance of the findings to the similar contexts of the study province only. Because all research was completed at one time point and due to seasonality of mosquito breeding and abundance this may have biased answers regarding responses on preventive behaviors.

## Conclusion

Successful dengue control requires an integrated approach following IVM principles and strong community participation with active roles for target communities. A well-informed and culturally appropriate COMBI strategy is required for sustained positive dengue prevention and control behaviors. The mix-media approach synchronizing the Interpersonal Communication (IPC) through volunteers, local media such as megaphones and songs and mass media such as radio and TV is vital to reinforce messages and ensures the information equity and reach to the target audience to achieve the behavioral changes. The current study demonstrated the perceived effectiveness and desire for the interventions (i.e. guppy fish, PPF, and COMBI activities). If shown efficacious through future entomological and epidemiological assessments they should be continued and further strengthened to ensure they are accessible, available and affordable as long as dengue continues to be a threat in these communities. These interventions should be further expanded or scaled up in order to extend the benefits of such initiatives to larger communities and better determine the effectiveness of such tools using epidemiological endpoints. The health volunteers are an effective and accepted channel of communication to engage communities, disseminate information and promote social and behavioral change by creating an enabling environment at the household and community level. There is a strong need to build the capacity of health volunteers in communication and community mobilization skills to ensure effective message delivery and active community participation in the interventions [43]. Given the potential for reducing dengue risk, further research into methods of achieving better guppy and PPF coverage and adoption in other communities should take place in the immediate future with a view to larger scale implementation.

## Supporting information

S1 File COREQ checklist for qualitative study. (DOCX)

S2 File Topic guides for focus group discussions and in-depth interviews. (DOC)

S1 Fig *Tuk Tuk*, a tool for communication for behavioral impact. (JPG)
