# Supplementary material for: Implementation of guppy fish (Poecilia reticulata), and a novel larvicide (Pyriproxyfen) product (Sumilarv 2MR) for dengue control in Cambodia: A qualitative study of acceptability, sustainability and community engagement
Source: PLoS Negl Trop Dis. 2019 Nov 18;13(11):e0007907. doi: 10.1371/journal.pntd.0007907 (PMC6886868; doi:10.1371/journal.pntd.0007907)
Supplement: S1 File — (DOCX) [file pntd.0007907.s001.docx]

**Table. Consolidated criteria for reporting qualitative studies (COREQ): 32-item checklist**

**No. Item Guide questions/description**

**………………………………………………………………………………………………………………..**

**Domain 1: Research team and reflexivity**

**Personal Characteristics**

| 1. Interviewer/facilitator | The FGD/IDI moderators were hired from a research agency. Keo Vanney, was the only research team member who moderated 4 FGDs which were triangulated with other facilitators’ data  **Page 1** |
| --- | --- |
| 1. Credentials | 1. Muhammad Shafique MA, 2. Sergio Lopes Msc, 3. Dyna Doum MPH, 4. Vanney Keo Msc, 5. Ly Sokha MD, 6. BunLeng Sam MD, 7. Chan Vibol MD, 8. Neal Alexander PhD, 9. John Bradley PhD, 10. Marco Liverani MSc, PhD, 11. Jeffrey Hii, PhD, 12. Leang Rithea MD, 13. Siddhi Aryal, PhD, 14. John Hustedt, MPH  **Page 1** |
| 1. Occupation | 1.Behavior Change Specialist, 2. Epidemiologist, 3. Senior Field Coordinator, 4. Field officer, 5. Program Manager, 6. Dengue Program Officer, 7. National Professional officer, 8. Professor of Medical Statistics and Epidemiology, 9. Assistant Professor of Medical Statistics and Epidemiology, 10. Lecturer in Health Policy, 11. Chief of Party – PMI Vectorlink Cambodia, 12. Cambodia Dengue Control Program Manager, 13. Asia Director, 14. Senior Technical Officer **Page 1** |
| 1. Gender | 4 male and 2 female participated in data collection  **Page 4** |
| 1. Experience and training | The data collectors had sufficient experience in qualitative research. A two-day refresher training was conducted on qualitative research methods, facilitation skills, probing skills, note taking skills, research ethics and topic guides  **Page 8** |
| **Relationship with participants** | |
| 1. Relationship established | Good rapport was developed before the interview to open up the participants and ensure quality discussion  **Page 8** |
| 1. Participants knowledge of the interviewers | Participants did not know the interviewers before the data collection as they were hired from different areas. Only few knew one interviewer (Keo Vanney)  **Page 8** |
| 1. Interviewers characteristics | Interviewers were hired from outside of the study areas and did not know the participants before the study.  **Page 8** |

**Domain 2: study design**

**Theoretical framework**

| 1. Methodological orientation and theory | Framework analysis  **Page 8** |
| --- | --- |
| **Participant selection** | |
| 1. Sampling | Purposive sampling technique was used to identify the study participants  **Page 7** |
| 1. Method of approach | Face to face interviews and focus group discussions were conducted at neutral and convenient places to ensure privacy and quality discussions  **Page 7,8** |
| 1. Sample size | 12 FGDs and 9 IDIs were conducted in which 103 persons participated  **Page 7** |
| 1. Non participants | No one refused to participate in the FGDs or IDIs. Only one male participant left in the middle of a FGD due to some personal urgency  **Page 4** |
| **Setting** | |
| 1. Setting of the data collection | Interviews were conducted at their households and FGDs were conducted at neutral common places, volunteer’s households and monasteries  **Page 8** |
| 1. Present of non-participants | Only participants were involved during the data collection. The interviews were conducted in private places to avoid the interference of non participants  **Page 8** |
| 1. Description of the sample | The participants were homogenized by age, gender, and occupation to ensure the quality of discussion  **Page 7** |
| **Data collection** | |
| 1. Interview guides | The main themes, questions and key probes were included in each topic to ensure in-depth discussion  **Page 7,8** |
| 1. Repeat interviews | No repeat interview was carried out |
| 1. Audio/visual recording | Audio recordings were collected of each interview and FGD with prior permission  **Page 8** |
| 1. Field notes | The detailed field notes were taken during the interviews to capture the key information, non-verbal behaviors and cues  **Page 8** |
| 1. Duration | Average duration of an FGD was 2 hours and IDI was 1 hour  **Page 8** |
| 1. Data saturation | The researcher held feedback sessions in the evening with data collector to discuss new emerging themes and saturation of data on daily basis  **Page 8** |
| 1. Transcripts returned | No, the transcripts were prepared by the data collectors and their were no major concerns to go back to communities for any to further confirmation  **Page 8** |

**Domain 3: analysis and findings**

| **Data analysis** |  |
| --- | --- |
| 1. Number of data coders | 1 person coded the data  **Page 8** |
| 1. Description of coding tree | The key themes, sub-themes were identified, however, no coding tree was developed.  **Page 8** |
| 1. Derivation of themes | Major themes/topics were identified in advance but the emerging themes and sub-themes were derived from the data on daily basis  **Page 9** |
| 1. Software | Excel spreadsheet was used for the data analysis  **Page 8** |
| 1. Participant checking | The data was triangulated by data collectors, methods i.e. interviews/FGDs and participants (gender). However, the participants were not involved in the data checking  **Page 7** |
| **Reporting** | |
| 1. Quotation presented | The participant quotations were presented to illustrate and validate the key findings. The quotes were identified by gender and intervention areas  **Page 8** |
| 1. Data and findings consistent | Yes, there were consistency between the data presented and findings  **Page 9-20** |
| 1. Clarity of major themes | Yes, the major themes were clearly presented in the findings  **Page 9-20** |
| 1. Clarity of minor themes | Yes, the minor themes were also clearly presented  **Page 17** |
