# Supplementary material for: Implementation of guppy fish (Poecilia reticulata), and a novel larvicide (Pyriproxyfen) product (Sumilarv 2MR) for dengue control in Cambodia: A qualitative study of acceptability, sustainability and community engagement
Source: PLoS Negl Trop Dis. 2019 Nov 18;13(11):e0007907. doi: 10.1371/journal.pntd.0007907 (PMC6886868; doi:10.1371/journal.pntd.0007907)
Supplement: S2 File — (DOC) [file pntd.0007907.s002.doc]

**Focus Group Discussion**

**Topic Guide for community members,**

**Kampong Cham, Cambodia**

**Intervention clusters**

FGD facilitator: …………………………………. FGD Note taker: ……………………… Date: ………………………………..

No. of participants: ………………………….. Time: ………………………………………. Village……………………………….

FGD Participant: (6 -8)

| **Major Themes** | **Questions/**Probes |
| --- | --- |
| General  (rapport building question) | What are the most common health problems in this community?Probe for all disease and dengue |
| Dengue | What do people call dengue in this community? Probe for all local terminologiesWhat are the common signs and symptoms of dengue? Probe for all signsIs dengue considered a serious health problem in this community? Why? Why not? |
| Cause of dengue | According to your opinion how do people get dengue? Causes of dengue? (Explore all existing beliefs)In which months of the year are people most likely to get dengue? Why, explore all reasonsWho are more at risk of dengue? Why |
| Key vector control measures  (Use free listing and liker scale exercise to animate the discussion) | What are the key methods you use to prevent dengue? Probe for all vector control measures?Free list all existing measures such as chemicals (abate, PPF, sprays), biological interventions (guppies, Bti), and environmental interventions (jar covers, treated net covers, etc.) |
| Preferences of vector control methods  Liker scale | Which vector control methods you preferred the most? Why? Liker scale exercise. Use the cards of various methods and rank them in three categories i.e. 1) like 2) neutral 3) dislike and probe for each reasons in details*Probe for all aspects i.e. access, availability, cost, convenient to use, cultural appropriateness, side effect issues, smell, based on the piles sorting etc. |
| Healthcare Seeking Behaviours | When a person suspect or gets dengue in your community what do the family members do for treatment?Probe for all types of health care providers including private practitioners, drugs shops, traditional healers, and faith healersHow long after fever starts do you seek care? What do you do when you are waiting for treatment?Who decides in the family where to seek treatment?What are the barriers to receive timely treatment for dengue? Probe for all the economic, cultural or other barriers to treatment seeking |
| IVM project | Have you heard about the IVM (guppy fish/PPF) project?Did you participate in any activity of the project? Which activities? Please explainWhat did the project overall achieve with regards to vector prevention and control at the household and community level? Please explain |
| Guppies  *Community’s perception*  *Barriers to use* | *The project introduced guppies and PPF in these communities, first I will ask your opinion about guppies:* How did you find the guppies intervention to prevent larvae? Please explain?Probe for their overall opinion about guppies i.e. effective, not effective, why?Is there anything which the community members did not like about guppies?Probe for all misperceptions and misbeliefs such as smell of guppies, contamination of water with guppies, etc.What were the main challenges you faced to use/maintain guppies in the water containers?Probe for all problems related to use, maintenance such as children play/kills guppies etc. |
| Pyriproxyfen (PPF)  *Community’s perception*  *Barriers to use* | *Now I would like to ask about the second intervention i.e. PPF (show a PPF matrix)* How did you find the PPF intervention? Effective? How? Explain, if not effective how, ExplainDo you have any issues/concerns of using PPF? Please explainProbe for any misperception/fear of side effect about the PPF use in the community?What were the main challenges you/your family faced to use/maintain PPF in your containers?Probe for issues such as using it in wrong sizes, wrong containers etc. |
| Guppies vs. PPF  *(only in guppy+PPF arm)* | If we compare both interventions, which intervention you found most effective? Why?Probe: if guppies, why and how effective they are? Explain with examplesIf PPF, how and why effective they are? Explain with examplesWhich intervention you liked/preferred the most? Please explainIf guppy, why? Please explainIf PPF, why? please explain |
| Cost /willingness to pay for guppy/PPF and other preferred interventions | What is the average cost you usually pay for the existing vector control measure you use?Probe for jar covers, guppy, abate, bti, bed net etc.What cost would you be willing to pay for guppy and PPF interventions?Probe for cost of guppy fishProbe for the PPFProbe for other intervention of their choice |
| COMBI | What are the main sources of information about health in this community?Probe: community health workers, health centre staff, village leaders, teachers, radio and TV etc.What communication sources the community trust or preferred most? Why?What do you think were the most preferred behaviour change (COMBI) activities of the IVM project? Why?Probe for Tuk Tuk songs and messages, the community made IEC materials and messages, health education by volunteers etc.Did community participate in the COMBI activities?If yes, which activities they participated?If no, why they did not participate? Please explain |
| Recommendation | What would you suggest to improve the use of these interventions (guppies/PPF) in the community? Please adviseWhat would you suggest to improve in our COMBI activitiesAnything else you would like to say/suggest THANK YOU VERY MUCH FOR YOUR PARTICIPATION |

**Focus Group Discussion**

**Topic Guide for community members**

**Kampong Cham, Cambodia**

**FOR CONTROL CLUSTERS**

FGD facilitator: …………………………………. FGD Note taker: ……………………… Date: ………………………………..

No. of participants: ………………………….. Time: ………………………………………. Village……………………………….

FGD Participant: (6 -8)

| **Major Themes** | **Questions/**Probes |
| --- | --- |
| General  (rapport building question) | What are the most common health problems in this community?Probe for all disease and dengue |
| Dengue | What do people call dengue in this community? Probe for all local terminologiesWhat are the common signs and symptoms of dengue? Probe for all signsIs dengue considered a serious health problem in this community? Why? Why not? |
| Cause of dengue | According to your opinion how do people get dengue? Causes of dengue? (Explore all existing beliefs)In which months of the year are people most likely to get dengue? Why, explore all reasonsWho are more at risk of dengue? Why |
| Key vector control measures  (Use free listing and liker scale exercise to animate the discussion) | What are the key methods you use to prevent dengue? Probe for all vector control measures?Free list all existing measures such as chemicals (abate, PPF, sprays), biological interventions (guppies, Bti), and environmental interventions (jar covers, treated net covers, etc.) |
| Preferences of vector control methods  Liker scale | Which vector control methods you preferred the most? Why? Liker scale exercise. Use the cards of various methods and rank them in three categories i.e. 1) like 2) neutral 3) dislike and probe for each reasons in details*Probe for all aspects i.e. access, availability, cost, convenient to use, cultural appropriateness, side effect issues, smell, based on the piles sorting etc. |
| Healthcare Seeking Behaviours | When a person suspect or gets dengue in your community what do the family members do for treatment?Probe for all types of health care providers including private practitioners, drugs shops, traditional healers, and faith healersHow long after fever starts do you seek care? What do you do when you are waiting for treatment?Who decides in the family where to seek treatment?What are the barriers to receive timely treatment for dengue? Probe for all the economic, cultural or other barriers to treatment seeking |
| Cost /willingness to pay for preferred interventions | What is the average cost you usually pay for the existing vector control measure you use?Probe for jar covers, guppy, abate, bti, bed net etc.Probe for other intervention of their choice |
| COMBI | What are the main sources of information about health in this community?Probe: community health workers, health centre staff, village leaders, teachers, radio and TV etc.What communication sources the community trust or preferred most? Why?Probe: Tuk Tuk songs and messages, IEC materials, health education by volunteers, anything else etc.Did community participate in any health education activities in the last 6 months?If yes, which activities they participated? Who organize these activities? How often they participated in these activitiesIf no, why they did not participate? Please explain |
| Recommendation | What would you suggest to improve the vector control activities in the community? Please adviseWhat would you suggest to improve in our existing behaviour change and community mobilization activitiesAnything else you would like to say/suggest THANK YOU VERY MUCH FOR YOUR PARTICIPATION |

**In-depth interview**

**Topic Guide for village leaders,**

**Kampong Cham, Cambodia**

FGD facilitator: …………………………………. FGD Note taker: ……………………… Date: ………………………………..

No. of participants: ………………………….. Time: ………………………………………. Village……………………………….

FGD Participant: (6 -8)

| **Major Themes** | **Questions/Probes** |
| --- | --- |
| General Question  Rapport building | - What do you think about dengue situation in these communities? - Probe for dengue cases, increasing or decreasing? Reasons for increase or decrease? |
| Key vector control measures | - What are the key vector control measures being used in the community? - Probe for all existing measures such as chemicals (abate, PPF, sprays), biological interventions (guppies, Bti), or environmental interventions (jar covers, treated net covers, etc.) and list of flip chart - Which vector control measure the community preferred the most and why? |
| Perception about IVM project | - What is your opinion about the IVM project? How did you find the project? explain - What did the project overall achieve with regards to vector prevention and control? please explain |
| Role and responsibilities (if any) | - What were your main roles and responsibilities in the project? Please explain - Probe for all activities such as guppy and PPF distribution, COBMI, health education |
| Guppies  *Perception*  *Barriers to use as volunteers* | *The project introduced guppies and PPF in these communities, first I will ask your opinion about guppies:*   - What is your opinion about the guppy intervention? Please explain? - Probe for their overall opinion about guppies i.e. effective, not effective, why? what they liked the most about the guppy intervention? Why? If they dislike anything, why? - What were the main challenges/difficulties you faced in supplying, distributing or maintaining guppies in these communities/volunteers? - Probe all challenges related to storage of guppies, supplies of guppies to communities/volunteers |
| Pyriproxyfen (PPF)  *Perception*  (ask only in the relevant clusters)  *Barriers to use/access* | *Now I would like to ask about the second intervention i.e. PPF (show a PPF matrix)*   - How did you as village leader find the PPF intervention? - Probe for the effectiveness of PPF as larvae/vector control intervention? What do you like the most about the PPF intervention? Why? If you dislike anything, what/why? - Probe for their perception i.e. side effects, fears of PPF or any other issue - What were the main challenges you faced in order to supply, store or maintain PPF at the community level? - Probe for all problems related to use, maintenance of PPF such as running short of the PPF, more demand from community/volunteers, shortage |
| Guppies vs. PPF  (Ask this question to clusters where both intervention took place) | - If we compare both interventions, in your opinion which is the most effective intervention for communities? - Probe: if guppies, why and how? Explain with examples - If PPF is effective, how and why? Explain with examples - According to your opinion/observations which intervention community liked the most? Please explain - If guppy, why? Please explain - If PPF, why? please explain |
| Sustainability of interventions | - What do you suggest for the continuous use of these interventions after the project closed? - Probe: how can we ensure that the communities will continue using the both guppies and PPF interventions after the project |
| COMBI | - What did the project achieve with regards to behavoural changes at the household and community level? - Probe for improvement in knowledge, behaviours of the community members regarding dengue prevention and control? - Probe for the changes in treatment seeking behaviours regarding dengue - What do you think were the most preferred COMBI/BCC activities of the project? Why? - Probe for Tuk Tuk songs and messages, the community made IEC materials and messages, health education by volunteers etc. |
| Community participation | - What do you think about the community participation in the project? Increased/decreased why? Please explain with examples - Which were the activities where community participation was very high? Why - Probe for the activities which received active community participation? - Which were the activities where community did not participate? Why |
| Recommendation | - How can we improve the use of these interventions (guppies/PPF) in the community? Please advise - What would you like to advise to improve our COMBI and community engagement intervention? - Anything else you would like to say |

**In-depth interview**

**Topic Guide for CNM/PHD/HC staff,**

**Kampong Cham, Cambodia**

FGD facilitator: …………………………………. FGD Note taker: ……………………… Date: ………………………………..

No. of participants: ………………………….. Time: ………………………………………. Village……………………………….

FGD Participant: (6 -8)

| **Major Themes** | **Questions/Probes** |
| --- | --- |
| General Question  Rapport building | - What do you think about dengue situation in these communities? - Probe for dengue cases, increasing or decreasing? Reasons for increase or decrease? |
| Existing vector control measures | - What are the key existing vector control measures available or being supported by the CNM, PHD or HC in these communities? - Probe for all existing measures such as chemicals (abate, PPF, sprays), biological interventions (guppies, Bti), or environmental interventions (jar covers, treated net covers, etc.) - Which vector control measure the institution (CNM, PHD or health center) prefers the most and why? - Probe for all aspects i.e. access, availability, cost, convenient to use, culturally appropriate, sustainability |
| Perception about IVM project | - What is your opinion about the IVM project? - What did the project overall achieve with regards to vector prevention and control? please explain |
| Role and responsibilities (if any) | - What were your main roles and responsibilities in the project? Please explain - Probe for all activities such as guppy and PPF distribution, COBMI, health education |
| Key vector control measures | - What are the key vector control measures available/being used in the community? - Probe for all existing measures such as chemicals (abate, PPF, sprays), biological interventions (guppies, Bti), or environmental interventions (jar covers, treated net covers, etc.) and list of flip chart - Which vector control measure the community preferred the most and why? |
| Guppies  *Perception*  *Barriers to use as volunteers* | *The project introduced guppies and PPF in these communities, first I will ask your opinion about guppies:*   - What is your opinion about the guppy intervention? Please explain? - Probe for their overall opinion about guppies i.e. effective, not effective, why? what they liked the most about the guppy intervention? Why? If they dislike anything, why? - What were the main challenges/difficulties you faced in supplying, distributing or maintaining guppies in these communities/volunteers? - Probe all challenges related to storage of guppies, supplies of guppies to communities/volunteers |
| Pyriproxyfen (PPF)  *Perception*  (ask only in the relevant clusters)  *Barriers to use/access* | *Now I would like to ask about the second intervention i.e. PPF (show a PPF matrix)*   - How did you as CNM, PHD or Health Centre staff find the PPF intervention? - Probe for the effectiveness of PPF as larvae/vector control intervention? What do you like the most about the PPF intervention? Why? If you dislike anything, what/why? - What were the main challenges you or your department (CNM, PHD, HC) faced in order to supply, purchase, store or maintain PPF at the community level? - Probe for all problems related to use, procurement, maintenance of PPF such as running short of the PPF, expensive, more demand from community/volunteers, shortage |
| Guppies vs. PPF  (Ask this question to clusters where both intervention took place) | - If we compare both interventions, in your opinion which is the most effective intervention for communities? - Probe: if guppies, why and how? Explain with examples - If PPF is effective, how and why? Explain with examples - According to your opinion/observations which intervention community liked the most? Please explain - If guppy, why? Please explain - If PPF, why? please explain |
| Sustainability of interventions | - What do you suggest for the continuous use of these interventions after the project closed? - Probe: how you or your institution can support the communities to continue using the both guppies and PPF interventions - What should we do to ensure the continuous use of these intervention after the project closed |
| COMBI | - What did the project achieve with regards to behavoural changes at the household level? - Probe for improvement in knowledge, behaviours of the community members regarding dengue prevention and control? - Probe for the changes in treatment seeking behaviours regarding dengue - What do you think were the most preferred COMBI/BCC activities of the project? Why? - Probe for Tuk Tuk songs and messages, the community made IEC materials and messages, health education by volunteers etc. |
| Community participation | - What do you think about the community participation in the project? Increased/decreased why? Please explain with examples - Which were the activities where community participation was very high? Why - Probe for the activities which received active community participation? - Probe for health education sessions, meetings? - Which were the activities where community did not participate? Why |
| Recommendation | - How can we improve the use of these interventions (guppies/PPF) in the community? Please advise - What would you like to advise to improve our COMBI and community engagement intervention? - Anything else you would like to say |
