# Supplementary figures and images for: Implementation of guppy fish (Poecilia reticulata), and a novel larvicide (Pyriproxyfen) product (Sumilarv 2MR) for dengue control in Cambodia: A qualitative study of acceptability, sustainability and community engagement
Source: PLoS Negl Trop Dis. 2019 Nov 18;13(11):e0007907. doi: 10.1371/journal.pntd.0007907 (PMC6886868; doi:10.1371/journal.pntd.0007907)

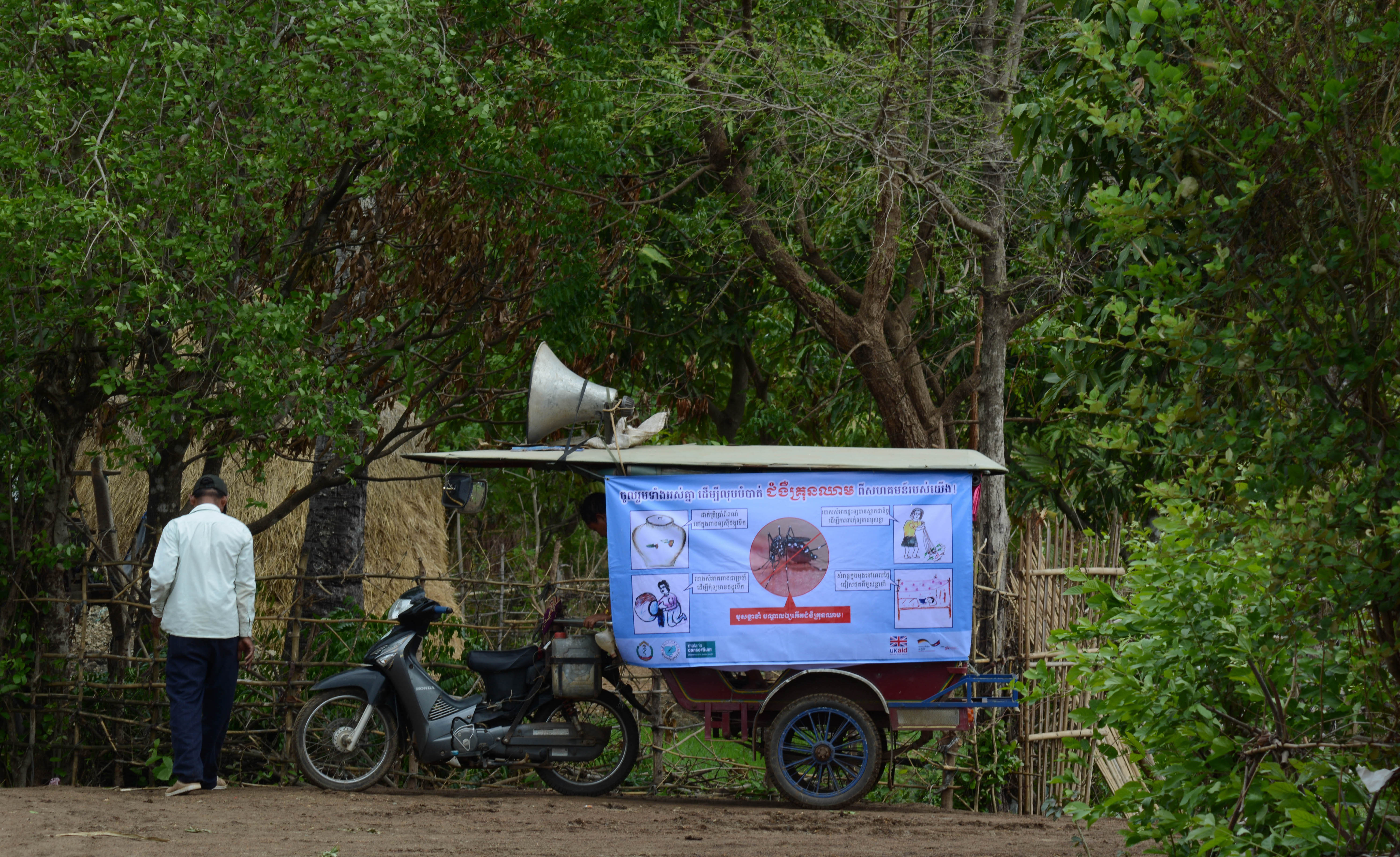

Supplement: S1 Fig — (JPG) [file pntd.0007907.s003.jpg]
